# Epigenetic Regulator miRNA Pattern Differences Among SARS-CoV, SARS-CoV-2, and SARS-CoV-2 World-Wide Isolates Delineated the Mystery Behind the Epic Pathogenicity and Distinct Clinical Characteristics of Pandemic COVID-19

**DOI:** 10.3389/fgene.2020.00765

**Published:** 2020-07-10

**Authors:** Md. Abdullah-Al-Kamran Khan, Md. Rabi Us Sany, Md. Shafiqul Islam, Abul Bashar Mir Md. Khademul Islam

**Affiliations:** ^1^Department of Mathematics and Natural Sciences, BRAC University, Dhaka, Bangladesh; ^2^Department of Genetic Engineering & Biotechnology, University of Dhaka, Dhaka, Bangladesh

**Keywords:** SARS-CoV-2, COVID-19, miRNA-microRNA, viral pathogenesis, immune regulation

## Abstract

A detailed understanding of the molecular mechanism of SARS-CoV-2 pathogenesis is still elusive, and there is a need to address its deadly nature and to design effective therapeutics. Here, we present a study that elucidates the interplay between the SARS-CoV and SARS-CoV-2 viruses' and host's miRNAs, an epigenetic regulator, as a mode of pathogenesis; and we explored how the SARS-CoV and SARS-CoV-2 infections differ in terms of their miRNA-mediated interactions with the host and the implications this has in terms of disease complexity. We have utilized computational approaches to predict potential host and viral miRNAs and their possible roles in different important functional pathways. We have identified several putative host antiviral miRNAs that can target the SARS viruses and also predicted SARS viruses-encoded miRNAs targeting host genes. *In silico* predicted targets were also integrated with SARS-infected human cell microarray and RNA-seq gene expression data. A comparison between the host miRNA binding profiles on 67 different SARS-CoV-2 genomes from 24 different countries with respective country's normalized death count surprisingly uncovered some miRNA clusters, which are associated with increased death rates. We have found that induced cellular miRNAs can be both a boon and a bane to the host immunity, as they have possible roles in neutralizing the viral threat; conversely, they can also function as proviral factors. On the other hand, from over representation analysis, our study revealed that although both SARS-CoV and SARS-CoV-2 viral miRNAs could target broad immune-signaling pathways; only some of the SARS-CoV-2 miRNAs are found to uniquely target some immune-signaling pathways, such as autophagy, IFN-I signaling, etc., which might suggest their immune-escape mechanisms for prolonged latency inside some hosts without any symptoms of COVID-19. Furthermore, SARS-CoV-2 can modulate several important cellular pathways that might lead to the increased anomalies in patients with comorbidities like cardiovascular diseases, diabetes, breathing complications, etc. This might suggest that miRNAs can be a key epigenetic modulator behind the overcomplications amongst the COVID-19 patients. Our results support that miRNAs of host and SARS-CoV-2 can indeed play a role in the pathogenesis which can be further concluded with more experiments. These results will also be useful in designing RNA therapeutics to alleviate the complications from COVID-19.

## Introduction

Coronavirus outbreaks have been reported over the past three decades, but the recent SARS-CoV-2 pandemic has reached more than 200 countries, has been the causative agent for the death of 58,392 people around the globe, and 1,087,374 coronavirus cases have been filed as of the date of writing this article (Worldometer, 2020). Among closed cases of SARS-CoV-2, 20% of the patients have died, and 5% of patients within active cases are in critical situations (Worldometer, 2020). The initial estimation of the SARS-CoV-2 death rate of 3.4%, as declared by the WHO (2020), requires refreshing, as the global casualty is on the rise. This novel virus requires novel and in-depth studies to promote new strategies for the management of this pandemic.

The coronavirus subfamily is a single-stranded positive-sense (+ssRNA) virus with a genome size of around 30 kb (Lu et al., 2020). The family is categorized into four subgenera as alpha, beta, gamma, and delta coronavirus (Cheng and Shan, 2020). SARS-CoV-2 is a beta coronavirus with a genome size of 29.9 kb (Accession no. NC_045512.2), 11 genes being reported in NCBI-Gene (2020). Phylogenetic analysis between SARS-CoV-2 and SARS-CoV showed ~79% similarity. Meanwhile, the distance is much greater for MERS-CoV (~50% similarity), but the closest relative to the SARS-CoV-2 is bat-derived SARS-like coronavirus (~90% similarity) (Jiang et al., 2020; Lu et al., 2020; Ren et al., 2020). Genomic analysis of SARS-CoV and SARS-CoV-2 has shown substitution of 380 amino acids and deletion of ORF8a, elongation of ORF8b (84 vs. 121 amino acid residues) and truncation of ORF3b (154aa in SARS-CoV whereas 22aa in SARS-CoV-2) (Lu et al., 2020).

MicroRNAs are small ncRNA molecules that regulate post-transcriptional-level gene expression; it has already been established that viruses use host machinery to produce miRNAs (Ambros, 2001). Although miRNA can be an important antiviral tool (Trobaugh and Klimstra, 2017) that can stimulate the innate and adaptive immune system (Ambros, 2001; Trobaugh and Klimstra, 2017) but that can also be a back door for viral propagation; it is non-antigenic, thereby modulating cellular pathways without triggering the host immune response (Cullen, 2013; Głobinska et al., 2014). For example, nucleocapsid protein of coronavirus OC43 binds miR-9 and activates NF-κB (Lai et al., 2014). Although host microRNAs are either utilized or regulated by viruses, viral miRNAs are another side of the coin; they regulate host gene expression, cellular proliferation, stress-related genes, and even viral gene expression (Cullen, 2010; Haasnoot and Berkhout, 2011; Lai et al., 2014). A summary explored how a number of DNA and RNA viruses produce miRNAs known as viral miRNAs (v-miRNAs) to evade the host immune response (Mishra et al., 2020). Novel viral miRNAs have been predicted to play an important role in neurological disorders as well (Islam et al., 2019). Among RNA viruses, HIV-1-encoded miR-H1, for example, can cause mononuclear cells apoptosis; H5N1 influenza virus-encoded miR-HA-3p targets host PCBP2 and contributes to the “cytokine storm” and mortality; and KUN-miR-1 of the West Nile virus targets the host's GATA4, which facilitates virus replication (Li and Zou, 2019). The interactions of host miRNAs with the SARS-CoV genome and viral proteins have been elucidated to suppress viral growth and immune evasion (Mallick et al., 2009). Novel classes of ncRNAs have been also observed by studies as possibly playing a definitive role in pathogenesis and survival (Liu et al., 2018). Respiratory viral infections caused by influenza, rhinovirus, adenovirus, RSV, and coronaviruses can be related to aberrant host miRNA expression, and their effect on the host can results in cell apoptosis, inhibition of immunologic pathways, downregulation of host antiviral responses, etc. (Mallick et al., 2009; Bondanese et al., 2014; Islam et al., 2019; Li and Zou, 2019; Mishra et al., 2020). Transmissible gastroenteritis virus (TGEV), though it induces significant IFN-I production after infection by inducing endoplasmic reticulum (ER), can evade antiviral effect of IFN-I by downregulating miR-30a-5p, which normally enhances IFN-I antiviral activity (Ma et al., 2018).

On the other hand, host miRNA expression plays a major role in controlling viral pathogenesis by mediating T cells and antiviral effector functions (Dickey et al., 2016). The first-reported example of a cellular miRNA that targets a viral RNA genome is miR-32, which targets the retrovirus PFV-1 transcripts and results in reduced virus replication (Lecellier et al., 2005). Similarly, miR-24, miR-93 can target VSV virus L and P proteins (Otsuka et al., 2007); miR-29a targets HIV Nef proteins (Ahluwalia et al., 2008) to inhibit replication; and miR-1, miR-30, miR-128, miR-196, miR-296, miR-351, miR-431, and miR-448 targets HCV C and NS5A proteins to inhibit translation/replication by inducing IFN signaling (Pedersen et al., 2007). miRNAs can therefore provide a different perspective in explaining the pathogenesis and infectivity of the novel SARS-CoV-2. Although SARS-CoV is distantly related to SARS-CoV-2, there are some similarities in their signs and symptoms, and they might be similar in pathogenesis, but there are crucial differences between the two diseases too (Xu et al., 2020). SARS-CoV-2 has infected many countries, and this has resulted in a stable mutation rate and some degree of variation (Xu et al., 2020). There is evidence that viral pathogens can have a novel immune evasion role by utilizing host miRNAs (Islam et al., 2019; Mishra et al., 2020).

**Figure 1:**
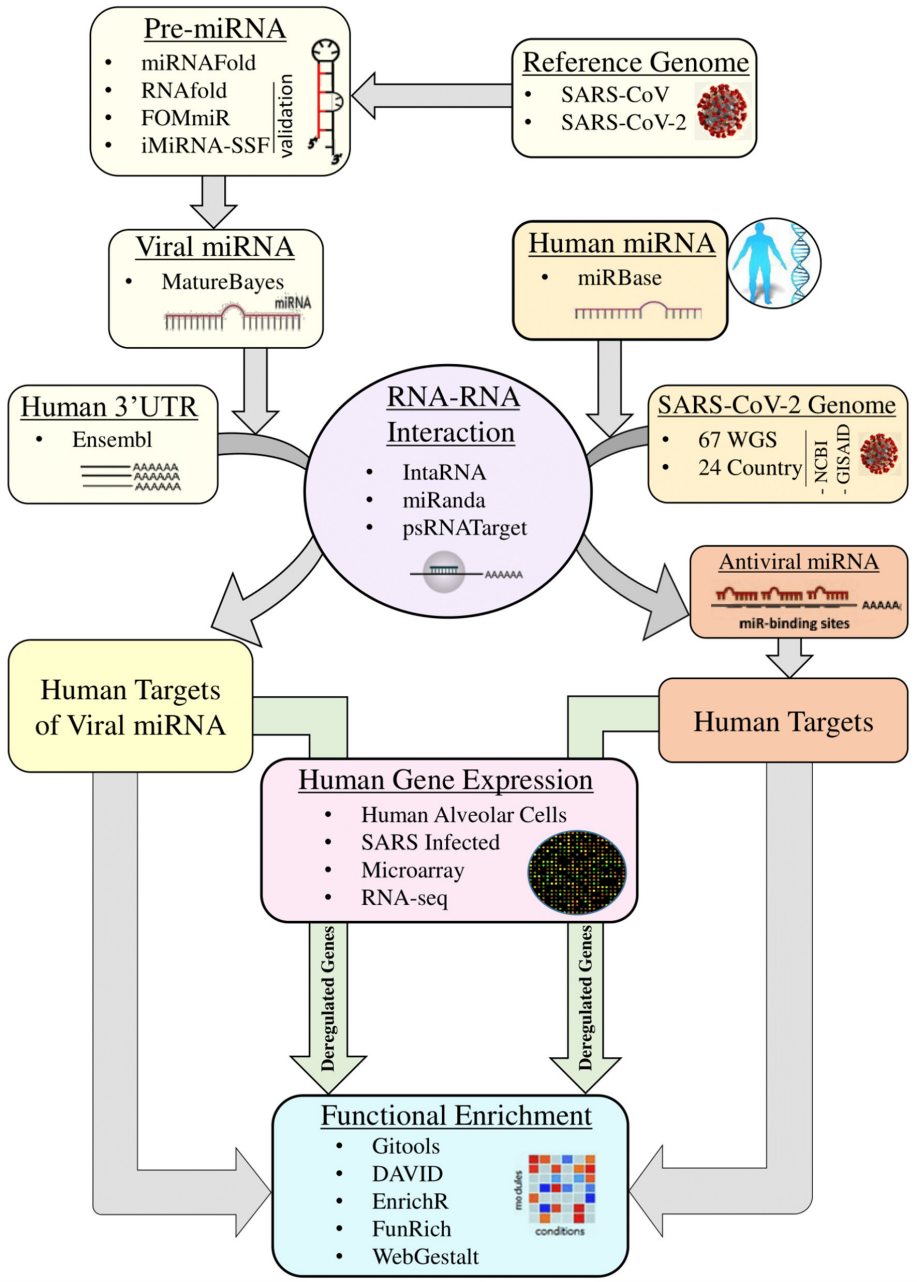
Overall workflow of the whole study.

The detailed miRNA-mediated epigenetic interplay between SARS-CoV-2 and its host is yet to be elucidated. It is not known what probable miRNAs produced by SARS-CoV-2 are affecting which human processes. Additionally, we do not know which antiviral miRNAs are taking part in host immunity. The genomic difference that, as a result, controls the host miRNA target sites and viral miRNAs might explain the difference between SARS-CoV and various isolated of SARS-CoV-2 in terms of pathogenesis and infectivity. Here in this study, we hypothesize on three potential effects of host and viral miRNA: (1) genomic differences between SARS-CoV and SARS-CoV-2 can lead to variations in host miRNA binding and differences in hence pathogenicity, signs and symptoms of these diseases and might explain the relatively longer incubation period of SARS-CoV-2; (2) on the other hand, there might be differences in viral miRNAs that can regulate expressions of different sets of host genes, which can in turn be advantageous to the virus or the host; and (3), due to a rapid mutation rate, observed variations among SARS-CoV-2 isolates in different regions of the world might result in variation in host capacities to target the virus with its miRNAs. This, in turn, might play a significant role in varying degrees of disease severity, symptoms, and mortality rate in different regions. In this study, we have carried out comparative analyses between SARS-CoV and SARS-CoV-2 with respect to host miRNA–viral genome interaction as well their differences based on region-specific isolates of SARS-CoV-2 and viral miRNA–host mRNA interactions to delineate the exclusive features of COVID-19 and their roles in viral survival and pathogenicity with respect to SARS-CoV (Figure 1).

## Materials and Methods

### Obtaining SARS-CoV and SARS-CoV-2 Genome Sequences

The reference genomes of SARS-CoV (RefSeq Accession no. NC_004718.3) and SARS-CoV-2 (RefSeq Accession no. NC_045512.2) were retrieved from the NCBI RefSeq database (NCBI-RefSeq, 2020). A total of 67 whole-genome sequences of SARS-CoV-2 isolates covering 24 different countries (Supplementary File 1) were retrieved from NCBI-Virus (2020) and Shu and McCauley (2017).

### Obtaining Human 3′UTR and Mature miRNA Sequences

Human miRNAs were accessed from microRNA database miRBase (Kozomara et al., 2018), and 3′UTR sequences of human protein-coding genes were obtained from Ensembl-Biomart (Hunt et al., 2018) (release 99).

### Prediction of Viral Pre-miRNA and Validation

We used miRNAFold (Tav et al., 2016) for *de novo* prediction of all possible precursor-miRNAs from the obtained reference sequences of SARS-CoV and SARS-CoV-2 with a sliding window size of 150 and minimum hairpin size as 0. The results were validated using three different tools. First, RNAfold (Gruber et al., 2008) was used with minimum free energy (MFE) and partition function fold algorithm to find stable secondary structures. Second, a fixed-order Markov model-based algorithm namely FOMmiR (Shen et al., 2012) was used. Finally, a SVM-based tool iMiRNA-SSF (Chen et al., 2016) was used that calculates minimum free energy (MFE), *p*-value of randomization test (*P*-value), and the local triplet sequence-structure features. The common predictions from these three tools were utilized for further analysis.

### Prediction of Mature miRNA

A Naive Bayes classifier algorithm implemented in tool MatureBayes (Gkirtzou et al., 2010) was used to identify mature miRNA candidates within the miRNA precursor sequences.

### RNA-RNA Interaction Analysis

Three different tools were used to analyze RNA–RNA interactions for the host miRNA-viral genome and viral miRNA-host 3′UTR of coding sequences. IntaRNA 2.0 (Mann et al., 2017) was used considering sites with parameters –mode=H, –model=X, –outMode=C, and ΔΔG ≤ −10 kcal/mol, with seed 2–8, allowing for G:U base pairs. microRNA.org (Betel et al., 2008) was used with a score cutoff ≥ 140, energy cutoff ≤ −20 kcal/mol, gap opening = −9.0, and gap extension = −4.0; psRNATarget (Dai and Zhao, 2011) with the default parameter was also used to determine RNA-RNA interactions. Finally, the common predictions from these three tools were considered for downstream analysis.

### Extraction of Targets of Host miRNAs

Targets of host miRNAs were mainly obtained using Funrich software (Pathan et al., 2017), which curates the experimentally validated targets of host miRNAs from different databases. The targets were also cross checked with the experimentally validated targets from miRTarBase database (Huang et al., 2019).

### Target Genes Functional Enrichment Analysis

#### Enrichment Analysis in Gitools

The functional annotation of target genes is based on Gene Ontology (GO) (Ashburner et al., 2000), as extracted from the EnsEMBL (Hubbard et al., 2007) and KEGG pathway database (Kanehisa and Goto, 2000). Accordingly, all genes are classified into the ontology categories' biological process (GOBP) and pathways when possible. We have taken only the GO/pathway categories that have at least 10 genes annotated. We used Gitools for enrichment analysis and heatmap generation (Perez-Llamas and Lopez-Bigas, 2011). Resulting *p*-values were adjusted for multiple testing using the Benjamini and Hochberg's method of False Discovery Rate (FDR) (Benjamini and Hochberg, 1995).

#### Enrichment Analysis Using Web-Based Tools

The host miRNAs targeting SARS-CoV and SARS-CoV-2 were used for functional over-representation analysis to visualize and predict the roles of these miRNAs in human diseases and find enriched pathways. Besides Gitools, functional enrichment analyses of the target human genes were conducted using EnrichR (Kuleshov et al., 2016), DAVID 6.8 (Huang et al., 2009; Sherman and Lempicki, 2009), WebGestalt 2019 (Liao et al., 2019), and FunRich 3.1.3 (Pathan et al., 2017). The targeted genes are analyzed to determine their role in viral pathogenesis, infectivity, and immune evasion.

### Microarray Expression Data Analysis

Microarray data for changes in gene expression induced by SARS-CoV on 2B4 cells, comparing the infected cells with SARS-CoV with the uninfected cells (for 12, 24, and 48 h), obtained from the Gene Expression Omnibus (GEO), ID GSE17400 (https://www.ncbi.nlm.nih.gov/geo) (Barrett et al., 2012). Raw Affymatrix CEL files were background corrected and normalized using the “rma” algorithm of Bioconductor package “affy” (version 1.28.1). The quality of microarray experiment (data not shown) was verified by Bioconductor package “arrayQualityMetrics” (Kauffmann et al., 2009) (version 3.2.4 under Bioconductor version 3.10; R version 3.6.0). To determine genes that are differentially expressed (DE) between two experimental conditions, Bioconductor package Limma (Smyth, 2005) was utilized to generate contrast matrices and fit the corresponding linear model. Probe annotations of genes were done using the Ensembl gene model (Ensembl version 99) as extracted from Biomart (Flicek et al., 2007) and by using in-house python script. When more than one probe was annotated to the same gene, the highest absolute expression value was considered (maximizing). To consider a gene is differentially expressed, multiple tests corrected FDR (Benjamini and Hochberg, 1995) *p*-value ≤ 0.05 was used as a cut-off.

### RNA-seq Expression Data Analysis

RNA-seq raw read-count data on SARS-CoV-2 mediated expression changes in primary human lung epithelium (NHBE), and transformed lung alveolar (A549) cells were obtained from the GEO database (GSE147507) (Barrett et al., 2012). For the differential expression (DE) analysis, we used the Bioconductor package DESeq2 (version 1.38.0) (Anders and Huber, 2010) with R version 3.6.0 (R Core Team, 2016) with a model based on the negative binomial distribution. To avoid false positives, we considered only those transcripts where at least 10 reads were annotated and a *p*-value of 0.01.

### MicroRNA Clustering

The hierarchal clustering of human miRNAs that could target SARS-CoV-2 genomes (binary mode) obtained from various countries was done using Manhattan distance and complete linkage analysis with the Genesis tool (Sturn et al., 2002). The human death count (per million population) due to SARS-CoV-2 infection was obtained on the 2nd of April, 2020, from the “Worldometer” website (Worldometer, 2020).

### Overlap Analysis

Two- or three-way overlap analysis was done using online venn-diagram program Venny 2.1.0 (Oliveros, 2018). Multiple pairwise overlaps, correlation analyses, and heatmap generations were carried out using Gitools (Perez-Llamas and Lopez-Bigas, 2011).

### Data Visualization

We have visualized human miRNAs that bind to the virus genome in web-genome browser NCBI genome data viewer (NCBI's-Genome-Browser, 2020).

## Results

### Several Human miRNAs Are Found to Target SARS-CoV and SARS-CoV-2

**Figure 2:**
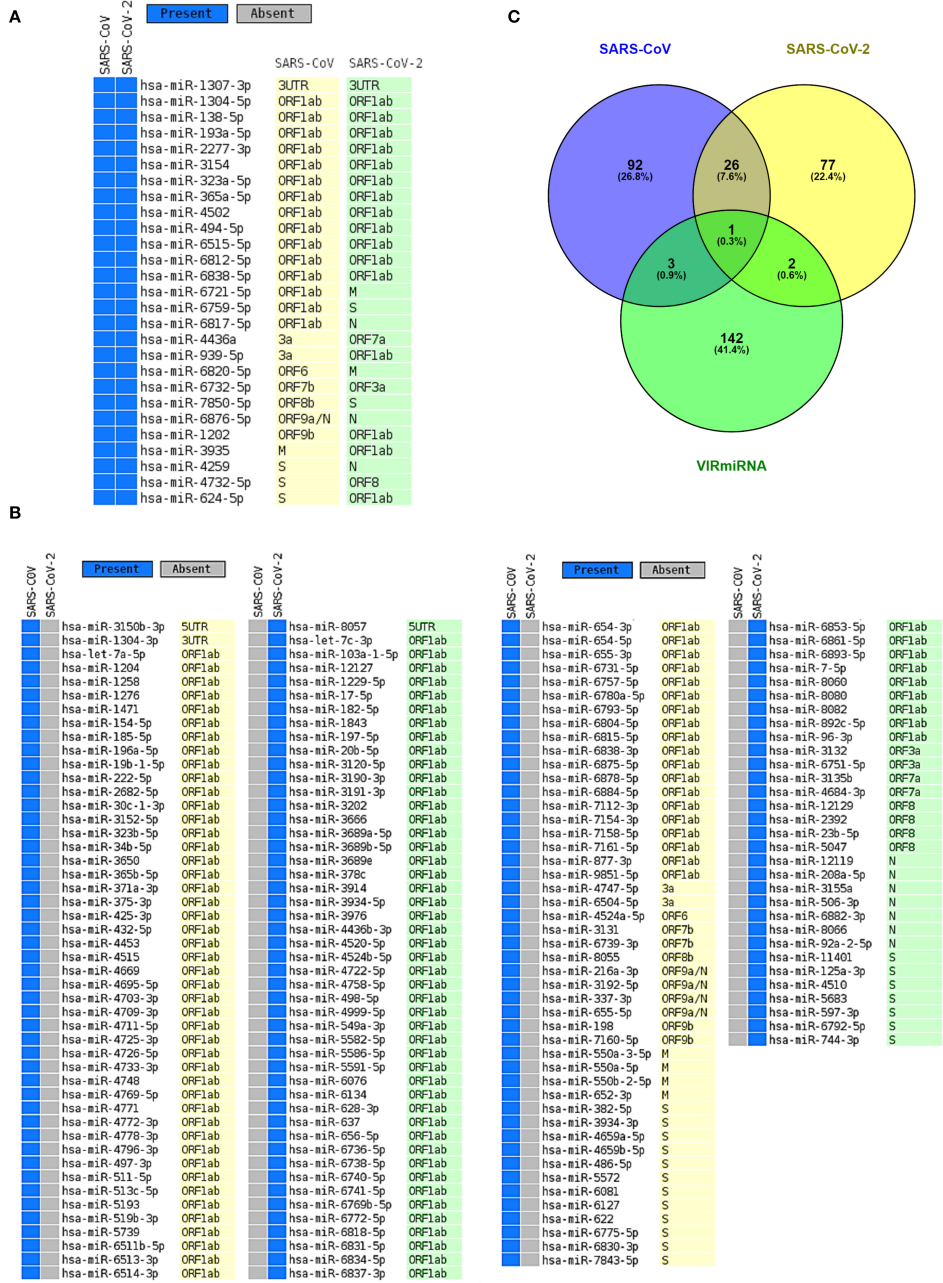
The host miRNAs targeting SARS-CoV and SARS-CoV-2. **(A)** Common host miRNAs and their target genes in SARS-CoV and SARS-CoV-2. **(B)** Host miRNAs and their target genes which uniquely target either SARS-CoV or SARS-CoV-2. **(C)** Venn diagram showing the common and unique host miRNAs targeting SARS-CoV and SARS-CoV-2 and host miRNAs that have exhibited experimental evidence as antiviral miRNAs.

It is possible that, during viral infections, host-encoded miRNAs can modulate viral infections as a means of a host immune response (Girardi et al., 2018). To identify possible host miRNAs that can get induced during the SARS-CoV (R) and SARS-CoV-2 (R) infections, we have utilized a bioinformatics approach. From our rigorous analysis pipeline, which covers three different well-established algorithms (IntaRNA, miRanda, and psRNATarget) to predict RNA-RNA interactions, we have identified 122 and 106 host antiviral miRNAs against SARS-CoV (R) and SARS-CoV-2 (R), respectively (Figures 2A,B, Supplementary File 2). Amongst these, 27 miRNAs were found to be targeting both viruses (Figure 2A). Whilst comparing these miRNAs with the antiviral miRNAs from VIRmiRNA (Qureshi et al., 2014), we have found four (hsa-miR-654-5p, hsa-miR-198, hsa-miR-622, and hsa-miR-323a-5p) and three (hsa-miR-17-5p, hsa-miR-20b-5p, and hsa-miR-323a-5p) host miRNAs against SARS-CoV (R) and SARS-CoV-2 (R), respectively, to exhibit experimental evidence of having antiviral roles during infections (Figures 2A–C).

**Figure 3:**
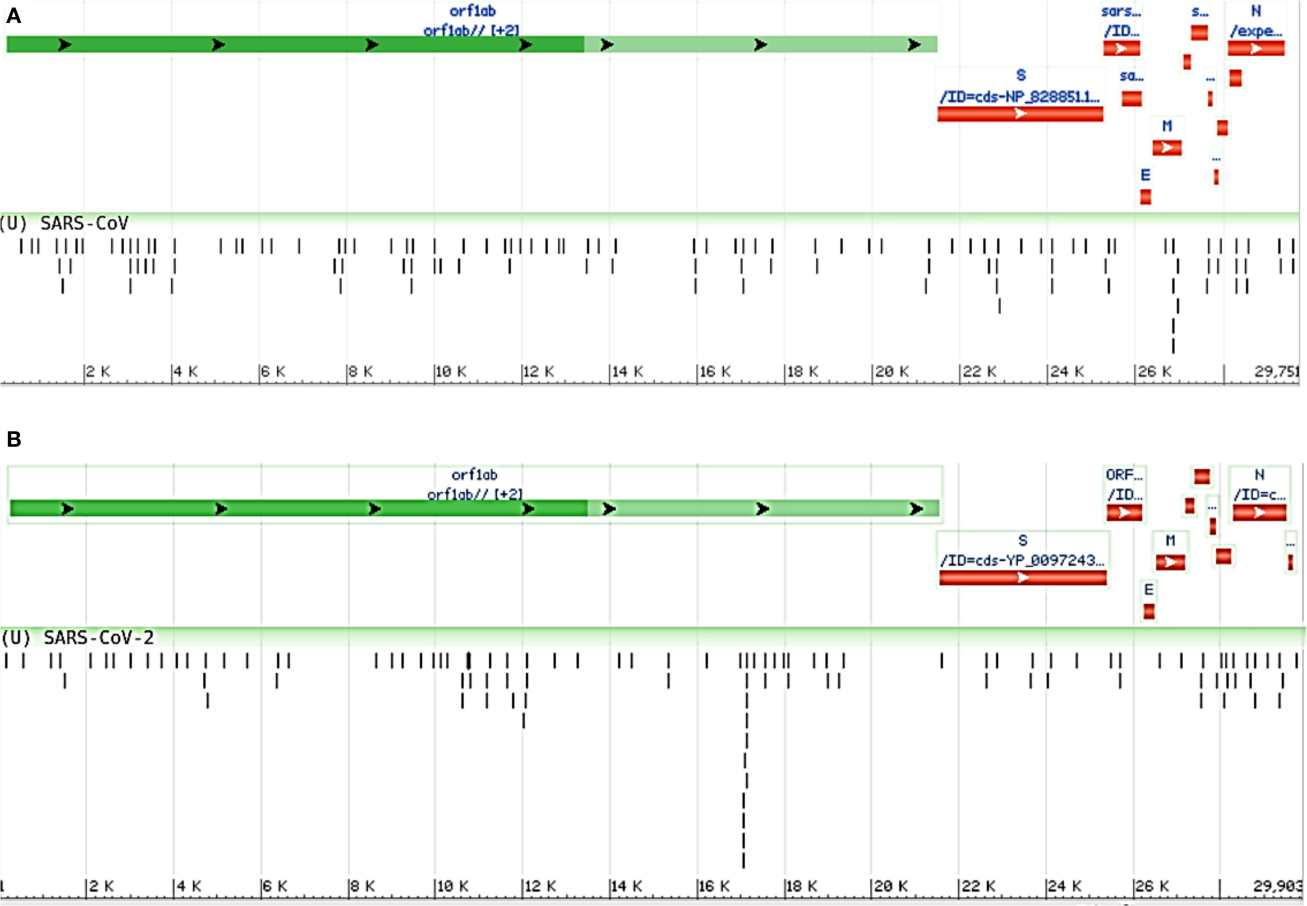
Genome browser view of host miRNAs targeting the regions of **(A)** SARS-CoV (Reference) and **(B)** SARS-CoV-2 (Reference) genomes.

Moreover, we compared the miRNAs targeting the two reference genomes of SARS-CoV (R) and SARS-CoV-2 (R), and we found most of the host miRNAs can target the ORF1ab region, followed by the S region as the second-most targeted (Figures 3A,B). Additionally, the M, N, ORF3a, ORF7a, ORF8 (ORF8a, ORF8b for SARS-CoV), 5′ UTR, and 3′ UTR regions of both viruses were targeted by host miRNAs. The significant variance was observed in the targeting positions of the host miRNAs between these two viruses (Figures 3A,B).

**Figure 4:**
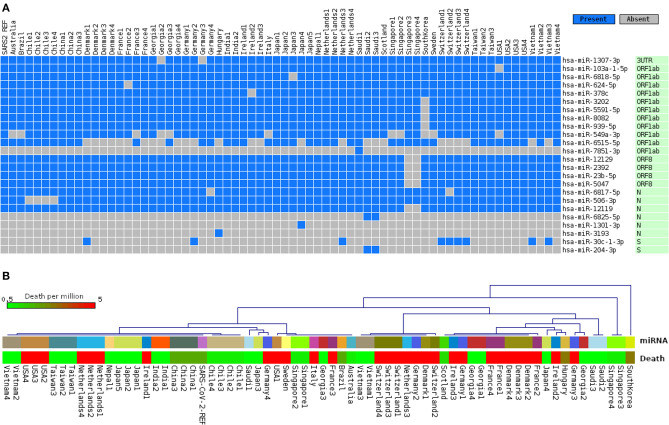
Differences of host miRNA-binding profiles, **(A)** representing only uncommon miRNAs binding pattern in 67 different SARS-CoV-2 genomes from 24 different countries, and **(B)** hierarchal clustering of all miRNAs binding in 67 genomes (upper panel, same country with same color code) and association of country-specific death rates (in color-coded scale) per million population (lower panel).

Since the RNA virus mutates fast, it is conceivable that mutations in crucial genomic locations would lead to differences in host miRNA binding patterns. Therefore, the ability of the host miRNAs in targeting genomes of 67 SARS-CoV-2 isolates covering 24 different countries was also seen. Although, as expected, most of the identified host miRNAs' binding profiles across these isolates remained somewhat similar to that of SARS-CoV-2 reference sequence; interestingly, we have identified 24 host miRNAs that bind differentially across the isolates (Figure 4A), which might have occurred due to the genomic variations between these isolates. Complete linkage agglomerative hierarchal cluster (HCL) analysis with Manhattan distance of these miRNAs (binary mode, bind or not bind) revealed two major clusters with a side cluster for one South Korean and two Singaporean isolates (Figure 4B). As miRNA is crucial in both host defense and viral pathogenesis, to understand the significance of this cluster, we have also compared the host miRNA clusters with the death rate (normalized by per million population) from different countries. Surprisingly, relatively higher deaths are found to be more prominent in the European major clusters (right side cluster) compared to the other major cluster (left side), and we also found much lower deaths in side clusters (Figure 4B). However, many more factors are there which can also play pivotal roles in the susceptibility of the patients, this suggests only one interesting aspect for this correlation between the miRNA binding pattern and host susceptibility.

### Host miRNAs Targeting SARS-CoV and SARS-CoV-2 Play Crucial Roles in Neutralizing the Virus

**Figure 5:**
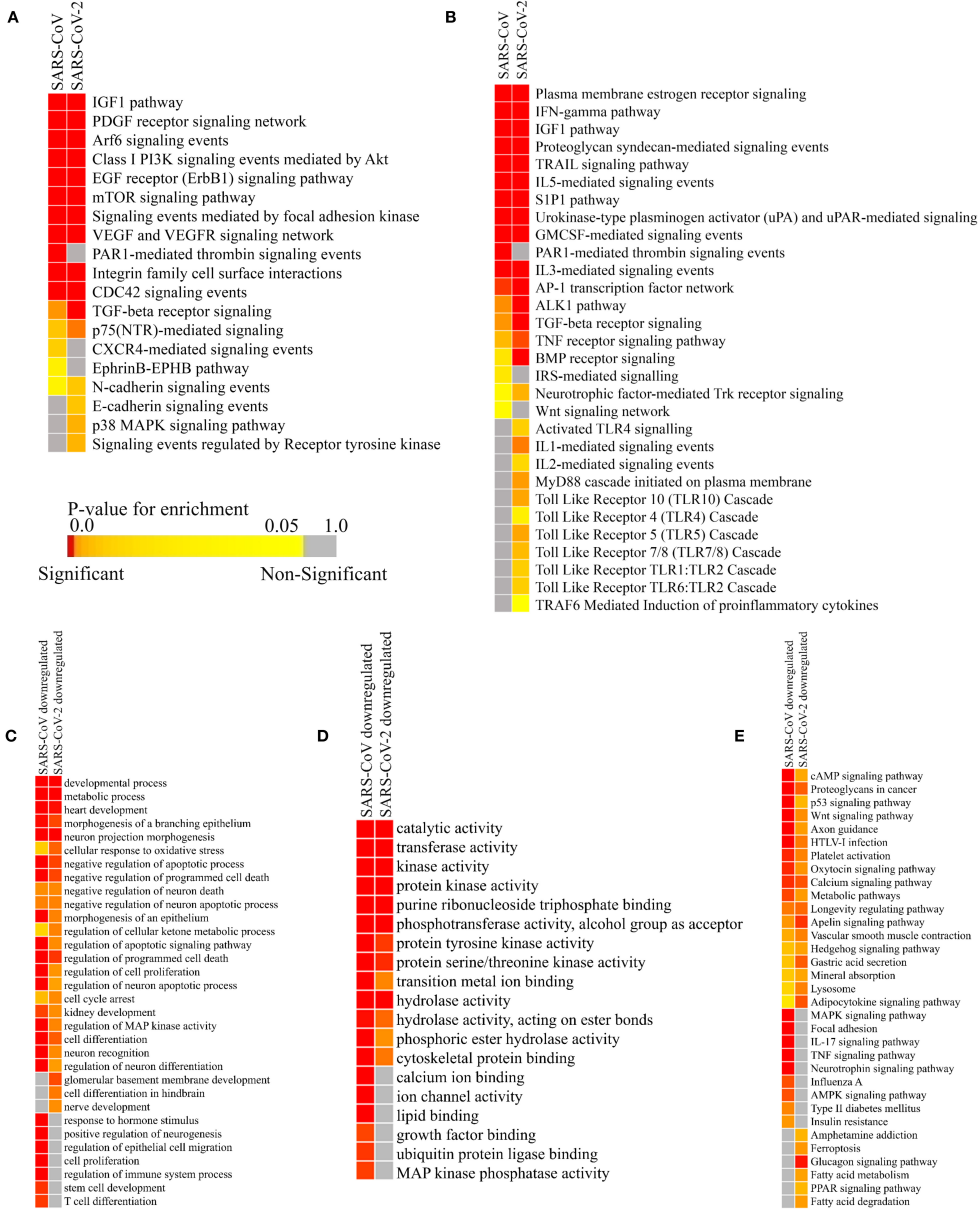
Enrichment analysis and comparison between host miRNA targets induced by SARS-CoV and SARS-CoV-2 infections. **(A)** Heatmap representation of enriched pathways involved in host defense obtained using Funrich software. **(B)** Enriched pathways that might act as proviral mechanisms obtained using Funrich software. Enrichment of downregulated host miRNA target genes in SARS-CoV and SARS-CoV-2 using gitools. **(C)** GO Biological Process module. **(D)** GO Molecular Function module. **(E)** KEGG pathway modules. The significance of enrichment in terms of adjusted *p*-value (<0.05) is represented in a color-coded *P*-value scale for all heatmaps. Color toward red indicates higher significance and color toward yellow indicates less significance, while gray means non-significant. Only selected significant enriched terms are shown.

Though the primary action elicited by host miRNAs is to silence the viral RNA, they might also modulate some host factors which provide an edge to the viral pathogenesis. To find out if these particular pathways are also targeted by the host miRNAs induced by SARS-CoV and SARS-CoV-2 infections, we have performed miRNA pathway enrichment analysis. We have found that several such pathways might be deregulated by the host miRNAs to suppress the entry of the virus, prevent the spread of the virions, and to minimize the systemic symptoms resulting from the infection (Figure 5A).

Host miRNAs might have a probable role in blocking the entry of the virus, as they are found to be targeting the pathways needed for viral entry- PDGF receptor-like signaling (Soroceanu et al., 2008), Arf-6 signaling (García-Expósito et al., 2011), PI3K-Akt signaling (Diehl and Schaal, 2013), EGFR signaling (Zheng et al., 2014), signaling events mediated by focal adhesion kinase (Elbahesh et al., 2014), CDC42 signaling (Swaine and Dittmar, 2015), the EphrinB-EPHB pathway (Wang et al., 2019), Cadherin signaling (Mateo et al., 2015), RTK signaling (Haqshenas and Doerig, 2019), etc. (Figure 5A).

They can also block some machinery like-p38 MAPK signaling (Hirasawa et al., 2003), FAK signaling (Elbahesh et al., 2014), PI3K-Akt signaling (Diehl and Schaal, 2013), etc., which can be hijacked by viruses for their efficient replication, pre-mRNA processing, and translation (Figure 5A). These host miRNAs might also try to reduce some host-induced inflammatory responses to prevent acute lung damage by targeting IGF1 signaling (Li et al., 2019), VEGF signaling (Alkharsah, 2018), PAR1 signaling (Heuberger and Schuepbach, 2019), integrin signaling (Teoh et al., 2015), TGF-beta signaling (Denney et al., 2018), TRAIL signaling (Cummins and Badley, 2009), etc. (Figure 5A). Some signaling pathways, such as CXCR4 signaling (Arnolds and Spencer, 2014), TGF-beta signaling (Denney et al., 2018), mTOR signaling (Le Sage et al., 2016), PI3K-Akt signaling (Diehl and Schaal, 2013), etc., can facilitate viral survival in infected cells by inhibiting apoptosis, autophagy, early immune responses, etc. Host miRNAs may function to downregulate these to invoke a proper immune response against the viruses (Figure 5A).

### Infection Induced Host miRNAs Can Function as a Proviral Factor by Inhibiting Host Immune Surveillance Pathways

Host miRNAs can be like double-edged swords, as sometimes, they can facilitate viral immune evasion by targeting some important host immune responses (Bruscella et al., 2017). Our host miRNA enrichment analysis showed several significant pathways, such as IFN-gamma signaling (Kang et al., 2018), TGF-beta signaling (Mogensen and Paludan, 2001), Interleukin signaling (Kimura et al., 2013), IGF1 signaling (Li et al., 2019), TRAIL signaling (Cummins and Badley, 2009), etc. These are involved in important proinflammatory cytokine signaling during viral infections (Figure 5B). Interestingly, we have found out that host miRNAs induced during SARS-CoV-2 infection may particularly downregulate the signaling of different Toll-Like Receptors (TLRs) (Kimura et al., 2013), which are considered as the primary stimulatory molecules for producing host antiviral responses (i.e., production of interferons and other inflammatory cytokines) (Figure 5B). Also, other receptor signaling involved in antiviral responses like- uPA-UPAR signaling (Alfano et al., 2003), TRAF6 signaling (Konno et al., 2009), S1P1 signaling (Oldstone et al., 2013), Estrogen receptor signaling (Kovats, 2015), Protease-activated Receptor (PAR) signaling (Antoniak et al., 2013), Bone morphogenetic protein (BMP) signaling (Eddowes et al., 2019), etc. can also be deregulated by the host miRNAs, leading to the host's immune suppression (Figure 5B).

### Host miRNAs' Targeted Downregulated Pathways Are Related to the Comorbidities of COVID-19

SARS-CoV-2-infected patients with comorbidities (i.e., cardiovascular diseases, diabetes, and renal problems) are found to be more susceptible to COVID-19. To find out whether host miRNAs play a role in these, we performed enrichment analyses using the downregulated targets genes of the host miRNAs using the expression data obtained from GEO dataset (GSE17400 for SARS-CoV and GSE147507 for SARS-CoV-2). These revealed that the downregulated targets of host miRNAs are involved in functions and pathways, such as heart development, kidney development, several neuronal processes, metabolic process, regulation of cellular ketone metabolism, insulin resistance, glucagon signaling pathway, fatty acid metabolism, and PPAR signaling (Figures 5C–E). Aberrant regulation of these processes can overcomplicate the disease conditions of patients having existing disorders.

### Viral miRNAs Encoded by SARS-CoV and SARS-CoV-2 Can Target Several Host Genes

**Figure 6:**
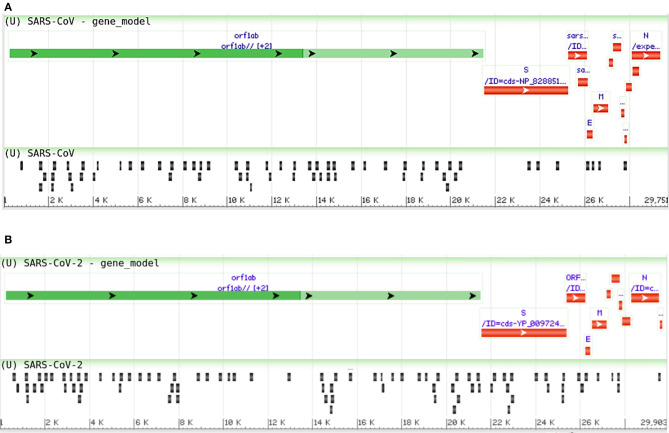
Genome browser view of viral miRNAs transcribed from the regions of **(A)** SARS-CoV (Reference) and **(B)** SARS-CoV-2 (Reference) genomes.

Many human viruses were found to produce miRNAs to assist in their overall pathogenesis by modulating host factors (Bruscella et al., 2017). Previous study on SARS-CoV also suggests that viral small non-coding RNAs can help its efficient pathogenesis (Morales et al., 2017). Our bioinformatics approach suggests that SARS-CoV and SARS-CoV-2 can also encode some viral miRNAs. The miRNAfold tool (Tav et al., 2016) yielded 529 and 519 putative pre-miRNAs from the genome of SARS-CoV and SARS-CoV-2, respectively. The RNAfold tool (Gruber et al., 2008) predicted 303 and 308 of these precursors of SARS-CoV and SARS-CoV-2, respectively are highly stable for forming hairpin structure which is a prerequisite of mature miRNA formation. Using FomMiR (Shen et al., 2012) and IMiRNA-SSF (Chen et al., 2016), we then predicted which of these highly stable precursors can truly produce mature miRNAs. We have found 63 and 85 such precursors, respectively for SARS-CoV and SARS-CoV-2. Using the Maturebayes tool from these precursors, we identified 126 and 170 mature miRNAs from SARS-CoV and SARS-CoV-2, respectively (Supplementary File 3). We predicted the human target genes by utilizing three different target prediction tools, and, reducing false positives, we have taken only the common set. This returned 5,292 and 6,369 human target genes for SARS-CoV and SARS-CoV-2, respectively (Supplementary File 4). Out of these, 2,992 genes are found to be common in both, while 2,300 and 3,377 genes were found to be unique targets of SARS-CoV and SARS-CoV, respectively. An apparent difference of the coding regions of miRNAs between SARS-CoV and SARS-CoV-2 was observed (Figures 6A,B).

### SARS-CoV and SARS-CoV-2 Can Evade Host's Immune Surveillance Pathway by Utilizing Its miRNAs

**Figure 7:**
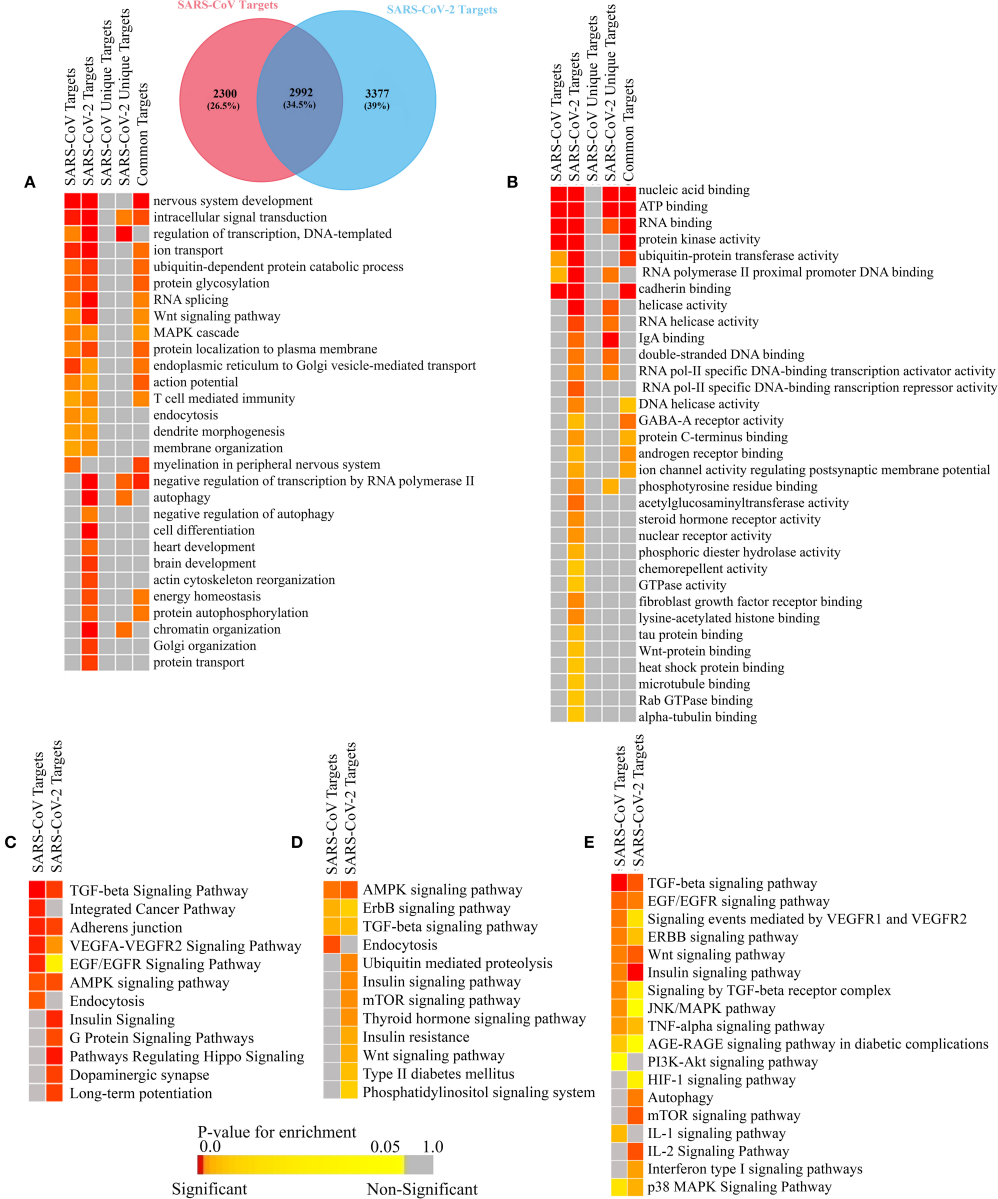
Enrichment analysis and comparison between the SARS-CoV and SARS-CoV-2 encoded viral miRNAs' target human genes. Functional enrichment using gitools: the **(A)** GO Biological Process module and **(B)** GO Molecular Funtion module. Enriched pathways obtained from **(C)** the Webgestalt (KEGG and Wikipathways) tool, **(D)** DAVID (KEGG pathways) tool, and **(E)** EnrichR (KEGG, Wikipathways, BioPlanet pathways) tool. Color codes are as in Figure 5. Only selected significant enriched terms are shown.

Many viruses use their miRNAs to suppress or escape host's immune responses (Mishra et al., 2020). To identify which pathways are associated with SARS-CoV and SARS-CoV infection, we have performed the gene ontology (GO) and pathway functional enrichment of the targeted genes using different tools. This revealed a myriad of significant functions and pathways involved in host immune responses, such as Wnt signaling (Ljungberg et al., 2019), MAPK signaling (Kimura et al., 2013), T-cell-mediated immunity (Channappanavar et al., 2014), autophagy (Yordy and Iwasaki, 2011), FGF receptor binding (van Asten et al., 2018), TGF-beta signaling (Denney et al., 2018), VEGF signaling (Alkharsah, 2018), ErbB signaling (Zheng et al., 2014), mTOR signaling (Le Sage et al., 2016), and TNF-alpha signaling (Kimura et al., 2013) are particularly targeted by SARS-CoV-2 (Figures 7A–E).

Functions and pathways, such as heart development, brain development, and the insulin signaling pathway, etc. (Figures 7A–E), were also enriched for SARS-CoV-2 only, which can be targeted by the viral miRNAs, making the patients with previous complications more susceptible to COVID-19, and it can also lead to several signs uniquely found in SARS-CoV-2-infected patients.

**Figure 8:**
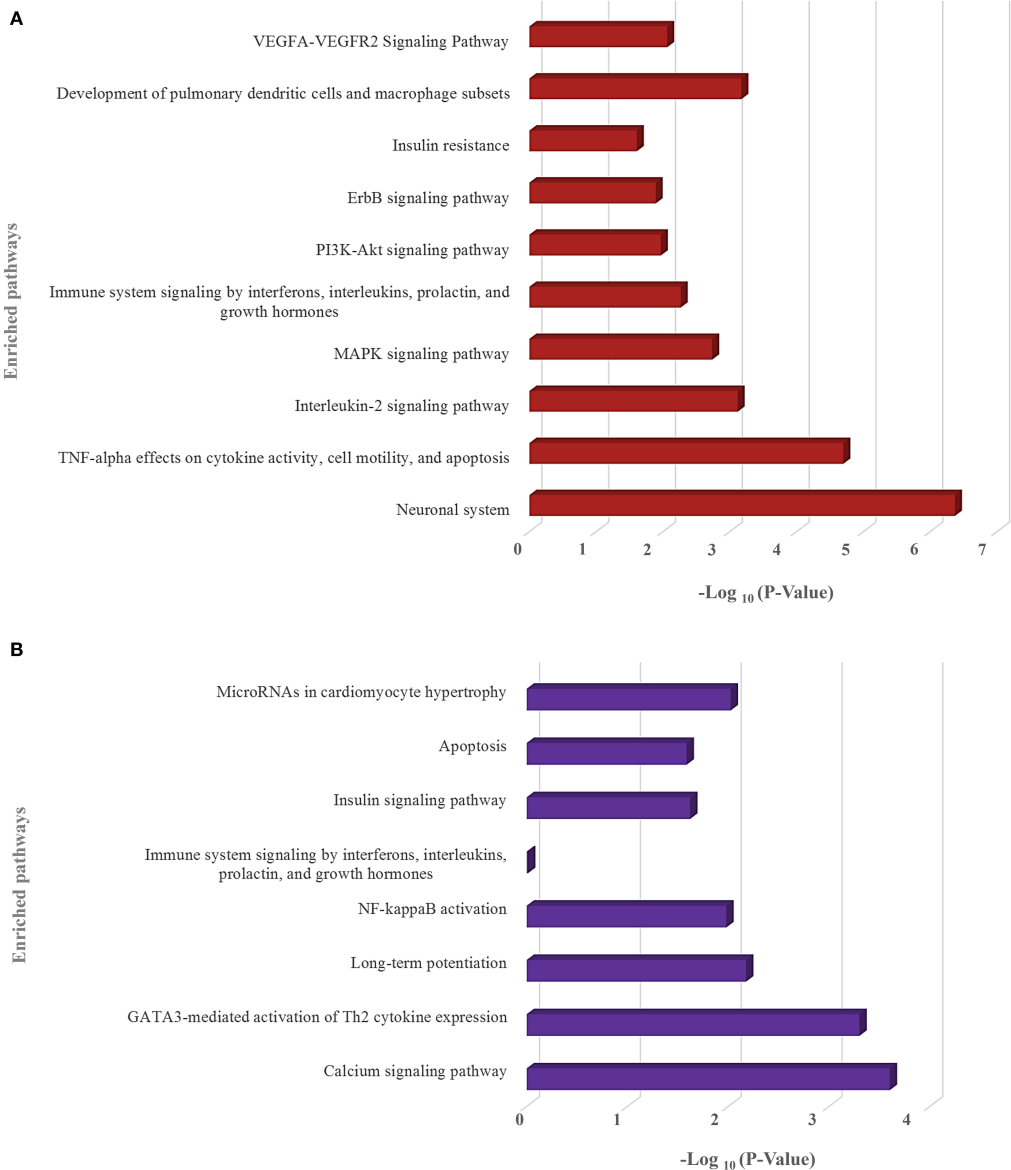
Enrichment analysis and comparison between the enriched pathways of **(A)** SARS-CoV and **(B)** SARS-CoV-2 encoded viral miRNAs' downregulated target genes, obtained using the EnrichR (KEGG, Wikipathways, and BioPlanet pathways) tool. –Log_10_ (*P*-value) scale is utilized for all in a bar graph. The higher the bar height, the more significant an enriched term is. Only selected significant enriched terms are shown.

We have also identified the downregulated target genes by curating the GEO expression datasets (GSE17400 for SARS-CoV and GSE147507 for SARS-CoV-2) and found 120 and 35 downregulated target genes in SARS-CoV and SARS-CoV-2, respectively (Supplementary File 5). These downregulated target genes are found to be involved in different immune-signaling pathways as well as different pathways related to organ-specific function (Figure 8).

## Discussion

Cellular miRNAs play a crucial role during the viral infection to strengthen host immunity by targeting virus genes as well as pathways that viruses utilize for their survival and immune evasion (Girardi et al., 2018). Viruses themselves can encode their miRNAs to target these immune-signaling pathways (Bruscella et al., 2017). COVID-19 has become a serious public health issue, though the complete molecular mechanism of pathogenesis is not fully understood yet. In this context, we have carried out this whole study to investigate the miRNA-mediated interactions between the host and the SARS-CoV-2 virus, which might shed some light on the tug-of-war between host's immune responses and virus's circumvention strategies. Though the disease conditions caused by SARS-CoV and SARS-CoV-2 are more or less similar, several unique features [i.e., long incubation, enhanced latency, asymptomatic infection, intense pain, severe lung damage, etc. (Ceccarelli et al., 2020)] of SARS-CoV-2 make it more challenging to manage compared to SARS-CoV. We also sought to find out if there are any existing differences between SARS-CoV and SARS-CoV-2 in the context of miRNA-mediated regulation of host responses.

As host miRNAs are one of the key sources of immune protection against viral infections, we have tried to find out which cellular miRNAs can target SARS-CoV and SARS-CoV-2 genes. Due to differences in the genome sequences between these two viruses, there was a significant difference between cellular miRNAs and their targeting viral genes. Likewise, some of the commonly found cellular miRNAs were showing differential binding preferences for these viral genes (Figure 2A). Previous study by Mallick et al. showed that cellular miRNAs can boost up host's immune response as well as they can assist in viral immune evasion mechanisms (Mallick et al., 2009). Another study by Morales et al. suggested that SARS-CoV can encode small non-coding RNAs that can play a role in inflammatory lung pathology (Morales et al., 2017). We identified that some of our predicted miRNAs have partial sequence similarities with the SARS-CoV svRNAs reported by Morales et al. (2017) (Supplementary File 6). We also compared the induced host miRNAs' profiles of 67 SARS-CoV-2 isolates from 24 different countries across the globe. From this analysis, we have identified several clusters and associated miRNAs, and our correlation study between these clusters with the death counts all over the world shed some light on the burning question and suggests why Europeans are more prone to COVID-19 (Figure 3B).

We found several miRNAs with experimentally validated antiviral roles; among those, hsa-miR-323a-5p and hsa-miR-654-5p (predicted for SARS-CoV) were found to inhibit viral replication in H1N1 Influenza virus infection (Song et al., 2010), while hsa-miR-17-5p and hsa-miR-20b-5p (predicted for SARS-CoV-2) were found to be upregulated in H7N9 Influenza virus infection (Zhu et al., 2014).

Apart from the basic role of cellular miRNAs in eliminating the transcripts of viruses, they can also modulate some host pathways which supposedly can be utilized by the infecting virus to avoid host's immune response. We also identified several such pathways involved in viral entry, replication, translation mechanisms, etc. These can be targeted by the cellular miRNAs induced by SARS-CoV and SARS-CoV-2 infection. Moreover, several immune-response pathways, such as TLR signaling, interleukin signaling, TRAF6 signaling, etc., were exclusively found to be targeted by SARS-CoV-2-induced host miRNAs (Figure 5B), and SARS-CoV-2-encoded miRNAs can target pathways, such as autophagy, IFN-I signaling, wnt signaling, mTOR signaling, etc. SARS-CoV-encoded miRNAs targets were, however, not found to be enriched in these pathways (Figures 7A–E). Target genes downregulated by SARS-CoV-2 miRNAs are found to be involved in the Ca^2+^ signaling pathway, and these are considered important activators of many signaling pathways (Zhou et al., 2009) (Figure 8B). All of these suggest why SARS-CoV-2 infections might be fatal for those who are immunosuppressed (D'Antiga, 2020).

Interestingly, our findings have enlightened several poorly understood mechanisms behind many of the unique clinical and pathological features of SARS-CoV-2, which has made it significantly different from SARS-CoV. We predicted both cellular miRNAs and viral encoded miRNAs, induced during SARS-CoV and SARS-CoV-2 infection, were found to target cytokine-signaling pathways involved in immune responses leading to the improved viral pathogenesis. Also, we found that SARS-CoV-2 miRNAs can target different important organ-specific cellular functions and pathways. We showed that SARS-CoV-2-encoded miRNAs can target the insulin-signaling pathway (Figure 7A, Supplementary Figure 1), and aberration of this pathway might overcomplicate the whole disease condition for COVID-19 patients with existing diabetic problems (Shimizu et al., 1980; del Campo et al., 2012). Our data also suggests that the SARS-CoV-2 miRNAs can target heart development-related pathways (Figure 7A, Supplementary Figure 1), which might lead to similar consequences like viral myocarditis (Dennert et al., 2008), making the disease more fatal for the patients with existing cardiovascular complications. These SARS-CoV-2-encoded miRNAs might also target genes associated with brain development (Figure 7A, Supplementary Figure 1), which might provide a clue about the neurological signs like headaches, vomiting, and nausea. SARS-CoV-2-induced host miRNAs can also downregulate kidney development and regulation of cellular ketone metabolic processes, etc. (Figure 5C), increasing the burden upon the kidneys (Kanikarla-Marie and Jain, 2016), which might be fatal for patients who have diabetes and kidney complications. HIF-1 signaling was also found to be targeted by SARS-CoV-2 miRNAs (Figure 7E, Supplementary Figure 1). This pathway is found to be associated with many viral infections, as HIF-1 plays an important role in cellular survival during hypoxic conditions (Santos and Andrade, 2017); COVID-19 patients suffer from the lack of oxygens due to breathing complications. This pathway might therefore play a crucial role in mitigating the condition, but viral miRNA-mediated deregulation of this pathway might result in severe consequences.

Our findings can explain that the interplay between host miRNAs and SARS-CoV-2 can promote viral pathogenesis by deregulating major antiviral immune-signaling pathways; furthermore, the resulting abnormal regulation of several host pathways might lead to an increased complications in the infected patients. Our study, which was conducted using machine learning and knowledgebase approaches, alongside further experiments, has the full potential to provide a more detailed understanding of the disease progression, and, based on these results, novel therapeutic interventions using RNA interference (RNAi) can be designed.

## Data Availability Statement

Publicly available datasets were analyzed in this study. This data can be found here: Gene Expression Omnibus (GEO), https://www.ncbi.nlm.nih.gov/geo/, Accession: GSE147507 and GSE17400.

## Author Contributions

AI conceived the project. AI and MK designed the workflow. MK, MS, and MI collected the data. All authors performed the analyses and wrote the manuscript. All authors contributed to the article and approved the submitted version.

## Conflict of Interest

The authors declare that the research was conducted in the absence of any commercial or financial relationships that could be construed as a potential conflict of interest.
