# Supplementary material for: Epigenetic Regulator miRNA Pattern Differences Among SARS-CoV, SARS-CoV-2, and SARS-CoV-2 World-Wide Isolates Delineated the Mystery Behind the Epic Pathogenicity and Distinct Clinical Characteristics of Pandemic COVID-19
Source: Front Genet. 2020 Jul 10;11:765. doi: 10.3389/fgene.2020.00765 (PMC7381279; doi:10.3389/fgene.2020.00765)
Supplement: Supplementary file 6 [file Data_Sheet_6.DOCX]

**Supplementary file 6:** Sequence overlap between our predicted SARS-CoV-encoded miRNAs and the svRNAs reported by Morales et al. (2017).

| **svRNA reported by Morales et al. (2017)** | **Start** | **End** | **Our predicted miRNA** | **Start** | **End** | **Max Score** | **Total Score** | **Query Coverage** | **E value** | **Percent Identity** |
| --- | --- | --- | --- | --- | --- | --- | --- | --- | --- | --- |
| nsp3.1 | 5 | 18 | 5'stem-miRNA 56 | 1 | 14 | 28.2 | 28.2 | 77% | 7e-05 | 100.00% |
| nsp3.2 | 8 | 17 | 5'stem-miRNA 56 | 1 | 10 | 20.3 | 34.7 | 59% | 0.022 | 100.00% |
|  | 12 | 18 | 5'stem-miRNA 437 | 8 | 14 | 14.4 | 14.4 | 31% | 1.3 | 100.00% |
| N | 2 | 9 | 3'stem-miRNA 251 | 14 | 21 | 16.4 | 16.4 | 38% | 0.32 | 100.00% |
|  | 1 | 7 | 3'stem-miRNA 259 | 4 | 10 | 14.4 | 14.4 | 33% | 1.3 | 100.00% |
|  | 14 | 20 | 3'stem-miRNA 208 | 13 | 19 | 14.4 | 28.7 | 38% | 1.3 | 100.00% |
|  | 1 | 7 | 5'stem-miRNA 437 | 10 | 16 | 14.4 | 14.4 | 33% | 1.3 | 100.00% |
