# Supplementary figures and images for: Epigenetic Regulator miRNA Pattern Differences Among SARS-CoV, SARS-CoV-2, and SARS-CoV-2 World-Wide Isolates Delineated the Mystery Behind the Epic Pathogenicity and Distinct Clinical Characteristics of Pandemic COVID-19
Source: Front Genet. 2020 Jul 10;11:765. doi: 10.3389/fgene.2020.00765 (PMC7381279; doi:10.3389/fgene.2020.00765)

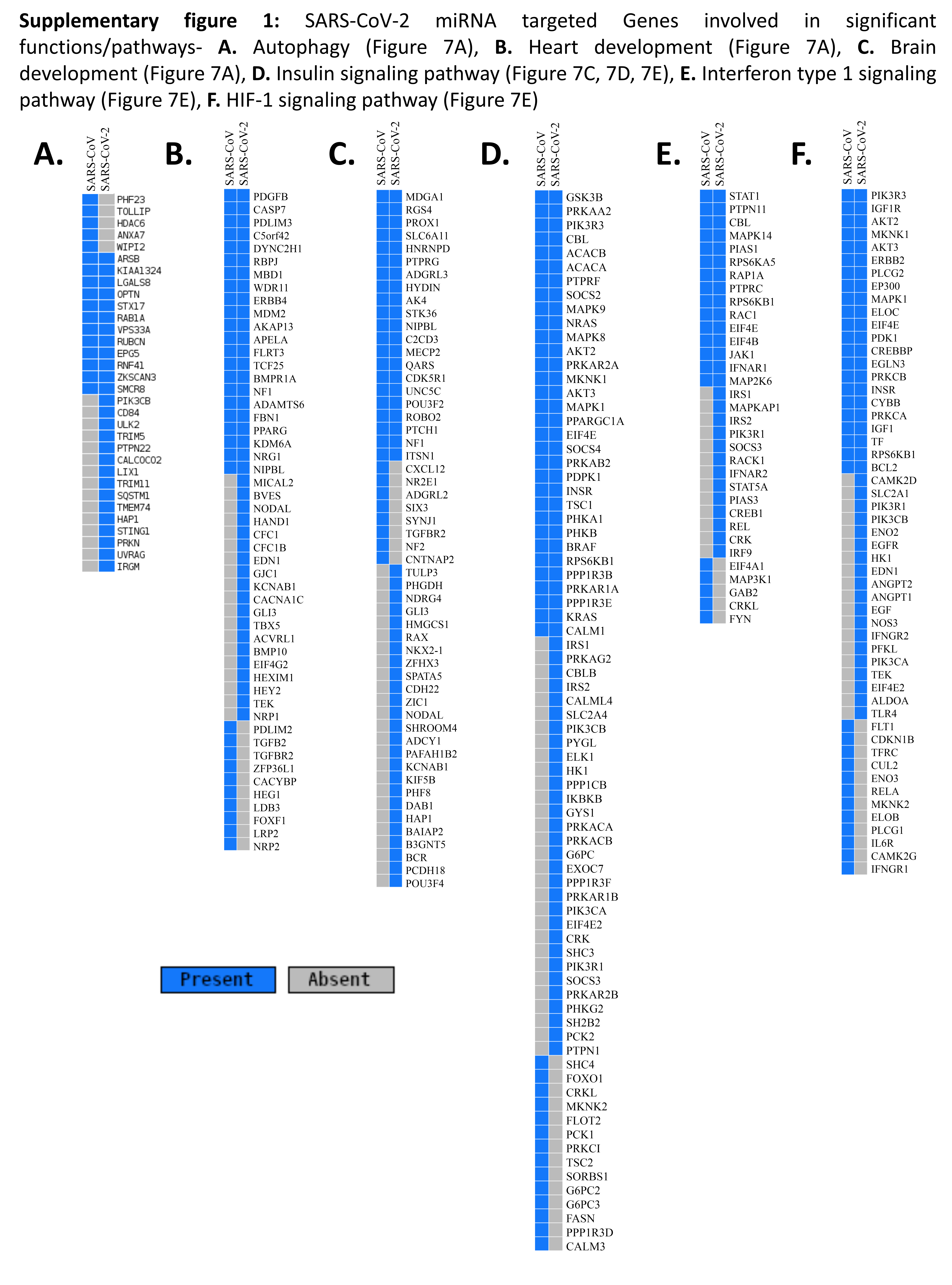

Supplement: Supplementary file 7 [file Image_1.TIF]
